# Sarcoidosis With Muscular and Peripheral Nervous System Involvement: An Atypical Presentation of a Rare Disease

**DOI:** 10.7759/cureus.103109

**Published:** 2026-02-06

**Authors:** Gonçalo Carneiro, Luís Fontão, Ricardo Taipa, Mafalda Santos, Ana Raquel Freitas

**Affiliations:** 1 Medicine, Unidade Local de Saúde de Entre Douro e Vouga, Santa Maria da Feira, PRT; 2 Neurology, Unidade Local de Saúde de Entre Douro e Vouga, Santa Maria da Feira, PRT; 3 Neuropathology, Unidade Local de Saúde de Santo António, Porto, PRT; 4 Internal Medicine, Unidade Local de Saúde de Entre Douro e Vouga, Santa Maria da Feira, PRT

**Keywords:** axonal polyneuropathy, granuloma, neurosarcoidosis, sarcoid myopathy, sarcoidosis

## Abstract

Neuromuscular sarcoidosis is uncommon and may represent a diagnostic challenge, particularly in the absence of more typical sarcoidosis manifestations such as pulmonary involvement. We report a case of a 71-year-old male presenting with constitutional symptoms, weight loss, progressive gait impairment, hypercalcemia, and acute kidney injury. Initial evaluation excluded malignancy, active infection, and monoclonal gammopathy. Advanced imaging showed diffuse muscular involvement, and muscle biopsy confirmed non-caseating granulomatous myositis, establishing the diagnosis of muscular sarcoidosis. The patient improved with corticosteroid therapy but developed sensory axonal polyneuropathy during tapering, requiring steroid-sparing immunosuppression with azathioprine. This case highlights the need to consider sarcoidosis in unexplained systemic and neuromuscular syndromes, even in the absence of pulmonary or other common extrapulmonary manifestations.

## Introduction

Sarcoidosis is a systemic granulomatous disease of unknown etiology, characterized by the formation of non-caseating granulomas in multiple organs [1]. Although pulmonary involvement is the most frequent manifestation (present in over 90% of cases), with ocular and cutaneous involvement being the most common extrapulmonary manifestations, neuromuscular sarcoidosis is rare. Neurosarcoidosis may present with peripheral neuropathy, cranial neuropathies, and small-fiber neuropathy, and is reported in approximately 5-15% of patients [2,3], while muscular sarcoidosis may manifest as sarcoid myositis and occurs in only a small minority of cases (<3%) [4]. These presentations may go unnoticed, be underdiagnosed, and lead to substantial morbidity and impaired quality of life [2-6].

This case aims to highlight an uncommon sarcoidosis phenotype, emphasizing the importance of including this diagnosis in the differential workup of patients with persistent systemic symptoms, particularly after exclusion of more common conditions and even in the absence of typical disease manifestations.

Explicit informed consent was obtained from the patient for publication of the clinical data and diagnostic imaging presented in this report.

## Case presentation

We report a case of a 71-year-old male with a history of arterial hypertension and degenerative lumbar spine disease, medicated with lisinopril, furosemide, and hydrochlorothiazide. He presented to the emergency department with unintentional weight loss of approximately 10 kg, asthenia, anorexia, and progressively worsening gait difficulties requiring walking aid support. Symptoms had been evolving over two months with gradual worsening. He denied other symptoms or relevant medical history, including familial or genetic diseases.

Physical examination revealed marked weight loss but no focal neurological deficits, lymphadenopathy, organomegaly, or abnormalities on cardiac and pulmonary auscultation.

Laboratory evaluation revealed acute kidney injury (creatinine = 2.2 mg/dL; baseline = 1.3 mg/dL) without associated proteinuria, hypercalcemia (ionized calcium = 1.87 mmol/L), low parathyroid hormone levels (1.5 pg/mL), and elevated angiotensin-converting enzyme levels (139.6 U/L). The autoimmune study was negative. A detailed summary of the initial laboratory findings is presented in Table 1, and the autoimmune and myositis-related antibody panel is shown in Table 2.

**Table 1:** Initial laboratory findings.

Test	Result	Reference range
Hemoglobin	13.5 g/dL	13.0–17.0
Mean corpuscular volume (MCV)	86.0 fL	80.0–100.0
Mean corpuscular hemoglobin (MCH)	32.4 pg	31.0–37.0
White blood cell count (WBC)	4,900/µL	4,000–11,000
Neutrophils (absolute)	3,230/µL	1,800–8,000
Lymphocytes (absolute)	930/µL	1,000–4,500
Platelet count	224,000/µL	150,000–450,000
Prothrombin time (PT)	11.9 s	—
International normalized ratio (INR)	1.0	—
Activated partial thromboplastin time (aPTT)	25.4 s	—
Urea	6.2 mg/dL	18–55
Creatinine	2.2 mg/dL	0.7–1.3
Total bilirubin	1.13 mg/dL	0.20–1.20
Aspartate aminotransferase (AST)	14 U/L	5–34
Alanine aminotransferase (ALT)	12 U/L	0–55
Alkaline phosphatase (ALP)	56 U/L	40–150
Gamma-glutamyl transferase (GGT)	13 U/L	<55
Lactate dehydrogenase (LDH)	146 U/L	125–220
Sodium	144 mmol/L	136–145
Potassium	4.3 mmol/L	3.5–5.1
Chloride	102.2 mmol/L	98–107
Ionized calcium	1.87 mmol/L	1.16–1.32
Phosphate	2.8 mg/dL	2.3–4.7
Magnesium	0.69 mmol/L	0.66–1.07
Erythrocyte sedimentation rate (ESR)	17 mm/h	0–35
C-reactive protein (CRP)	39.9 mg/L	<5.0
Total protein	5.4 g/dL	6.4–8.3
Albumin	2.6 g/dL	3.4–4.8
Thyroid-stimulating hormone (TSH)	2.27 µIU/mL	0.35–4.94
Free thyroxine (Free T4)	13.3 pmol/L	9.0–19.0
Parathyroid hormone (PTH)	1.5 pmol/L	1.6–7.2
Creatine kinase (CK)	54 U/L	30–200
Myoglobin	232.1 ng/mL	<140.0
Immunoglobulin A (IgA)	429 mg/dL	101–645
Immunoglobulin G (IgG)	1,093 mg/dL	540–1,822
Immunoglobulin M (IgM)	60 mg/dL	22–240
Free light chains (kappa/lambda)	287 mg/dL; 155 mg/dL	122–437; 62–231
β2-microglobulin	15.77 mg/L	<3.0
Prostate-specific antigen (PSA)	1.23 ng/mL	<4
Angiotensin-converting enzyme (ACE)	139.6 U/L	13.3–63.9
Urinalysis	No abnormalities	—
Serum protein electrophoresis	Normal pattern	—
- Albumin	49.9% (2.69 g/dL)	4.02–4.76
- Alpha-1 globulin	5.2% (0.28 g/dL)	0.21–0.35
- Alpha-2 globulin	11.0% (0.59 g/dL)	0.51–0.85
- Beta globulin	12.8% (0.69 g/dL)	0.60–0.94
- Gamma globulin	21.1% (1.14 g/dL)	0.80–1.35
- Albumin/Globulin ratio (A/G ratio)	1.00	—
Urinary immunofixation electrophoresis	No monoclonal fractions were detected	—

**Table 2:** Autoimmune and myositis-related antibody panel.

Test	Result	Reference range
Antinuclear antibodies (ANA)	0.4 U/mL	(<0.7)
Rheumatoid factor	<20 IU/mL	(<30)
Complement C3/C4	83/30 mg/dL	(82–185/15–53)
ANCA anti-MPO	0.10 IU/L	(<3.5)
ANCA anti-PR3	0.3 IU/mL	(<2)
Anti-Jo-1	Negative	—
Anti-SRP	Negative	—
Anti-Mi-2	Negative	—
Anti-PL7	Negative	—
Anti-PL12	Negative	—
Anti-EJ	Negative	—
Anti-OJ	Negative	—
Anti-Ku	Negative	—
Anti-PM-Scl100	Negative	—
Anti-PM-Scl75	Negative	—
Anti-Ro-52	Negative	—

Renal ultrasound revealed diffuse increased renal parenchymal echogenicity with reduced corticomedullary differentiation. Cervical, thoracic, abdominal, and pelvic computed tomography showed no significant abnormalities. Chest wall and skull radiographs, upper gastrointestinal endoscopy, and total colonoscopy were also performed, all without clinically significant findings.

The patient was admitted to an intermediate care medical unit and was treated with intravenous 0.9% sodium chloride at 200 mL/h and intravenous loop diuretic therapy (furosemide 10 mg every eight hours) for three days, as well as a single 4 mg dose of intravenous zoledronic acid. Renal function recovered to baseline, and hypercalcemia was corrected, although without significant clinical improvement. After discharge, he remained under follow-up in the internal medicine outpatient clinic.

**Figure 1:**
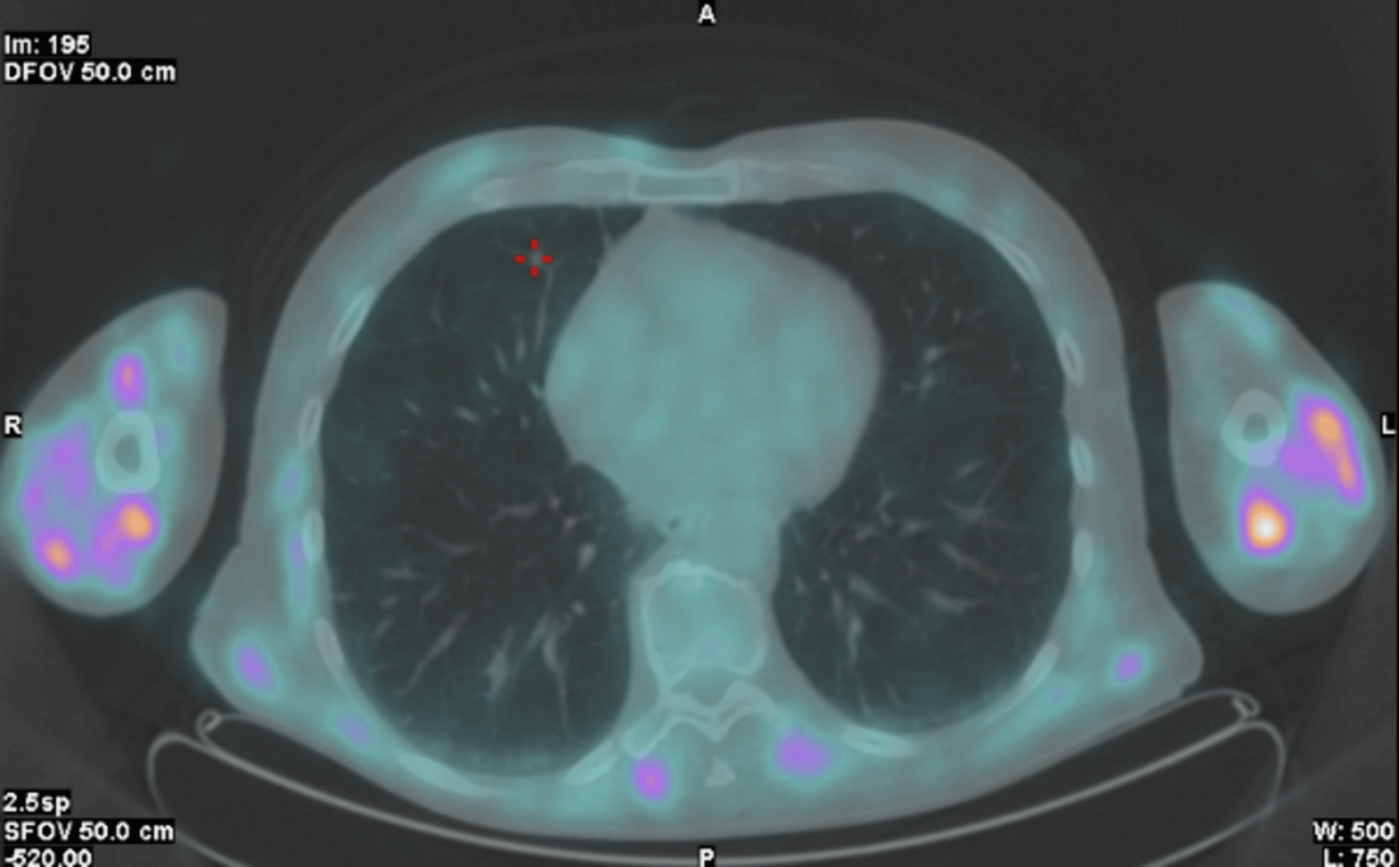
“Tiger man sign" on 18F-FDG PET/CT (axial fused image at the thoracic level), showing bilateral and symmetric hypermetabolic striated uptake in the skeletal muscles of the upper limbs, with no significant thoracic FDG-avid lesions. FDG: fluorodeoxyglucose.

**Figure 2:**
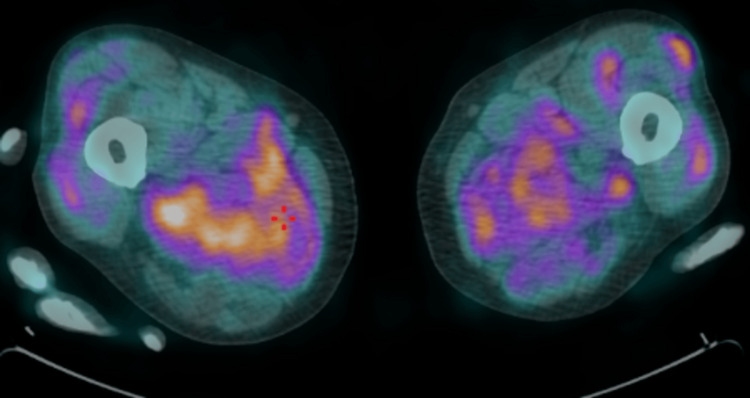
“Tiger man sign" on 18F-FDG PET/CT (axial fused image at the thigh level), demonstrating diffuse, heterogeneous, and fascicular-pattern FDG uptake in the skeletal muscles of both lower limbs. FDG: fluorodeoxyglucose.

Given the absence of a definitive diagnosis after exclusion of malignancy, active infection, and monoclonal gammopathy, an 18F-fluorodeoxyglucose (18F-FDG) positron emission tomography (PET) scan was performed, which revealed diffuse and generalized muscular uptake, a finding described as the "tiger man sign" (Figures 1, 2).

**Figure 3:**
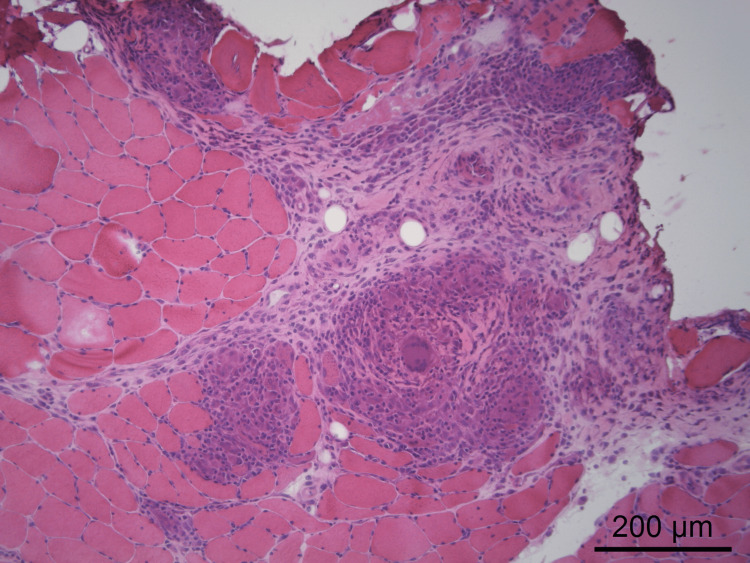
Muscle biopsy showing non-necrotizing granulomas accompanied by numerous multinucleated giant cells (hematoxylin and eosin staining).

**Figure 4:**
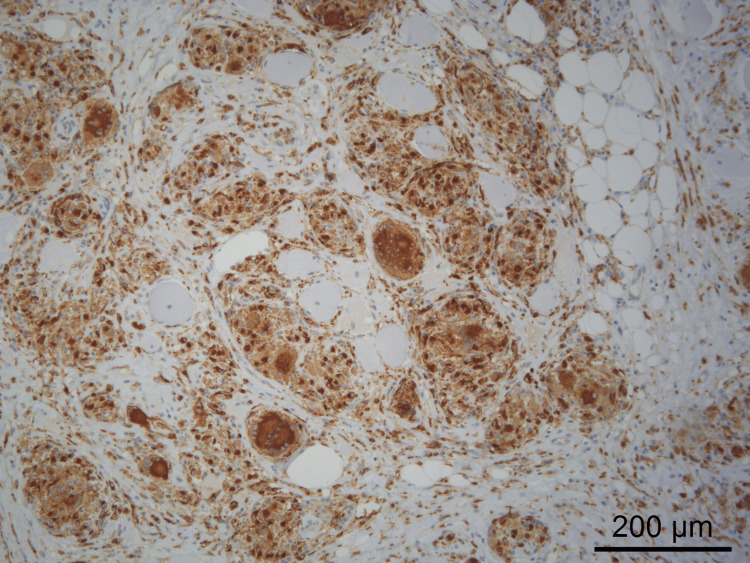
Muscle biopsy showing non-necrotizing granulomas accompanied by numerous multinucleated giant cells (CD68 immunohistochemistry).

In this context, a myositis-specific and myositis-associated autoantibody panel was requested and was negative (Table 2), and a muscle biopsy was performed, showing findings compatible with inflammatory myopathy, with the presence of non-necrotizing granulomatous infiltrates containing multinucleated giant cells (Figures 3, 4), thus establishing the diagnosis of muscular sarcoidosis.

Treatment with oral prednisolone at 1 mg/kg/day (80 mg/day) resulted in marked clinical improvement. Prednisolone was maintained at this dose for one month, followed by tapering at 10 mg/week until 10 mg/day. During corticosteroid tapering, the patient developed paresthesia and hypoesthesia of the lower limbs. Electromyography showed findings consistent with a symmetric distal-to-proximal sensory axonal polyneuropathy. After multidisciplinary discussion with the neurology team and the autoimmune disease team, peripheral nervous system involvement was confirmed, and azathioprine was initiated as a steroid-sparing agent (25 mg/day for two weeks, increased to 50 mg/day, and subsequently titrated up to 100 mg/day), allowing further corticosteroid tapering to discontinuation while maintaining clinical improvement.

## Discussion

The initial presentation with nonspecific constitutional symptoms prompted extensive investigation to exclude more prevalent diagnoses in this age group, particularly malignancy. Suppressed parathyroid hormone (PTH) levels ruled out primary hyperparathyroidism, and normal serum and urine electrophoresis, together with normal immunoglobulin levels, the absence of radiological lytic bone lesions, and no abnormal bone uptake on PET/CT, made monoclonal gammopathies, particularly multiple myeloma, unlikely, suggesting a non-PTH-dependent mechanism for hypercalcemia. Although β2-microglobulin was markedly elevated, this finding is nonspecific and must be interpreted in context. Serum β2-microglobulin can increase in conditions associated with reduced glomerular filtration and systemic inflammation, as it is filtered and cleared by the kidneys, and reflects immune activation [7]. Therefore, in the presence of normal electrophoresis studies and no evidence of osseous involvement, β2-microglobulin lacks specificity as a standalone marker for plasma cell disorders [7].

Although ACE levels have limited sensitivity and specificity, their elevation, together with persistent systemic symptoms and the etiological workup performed up to that point, kept the possibility of an underlying granulomatous disease open and supported the decision to pursue advanced diagnostic modalities [8].

Accordingly, 18F-FDG PET/CT was performed and demonstrated the "tiger man sign," characterized by diffuse, bilateral, and symmetric skeletal muscle uptake with a heterogeneous and striated appearance. This pattern is thought to reflect multifocal granulomatous inflammation distributed along muscle fascicles and interstitial tissue, leading to patchy areas of increased glucose metabolism interspersed with relatively spared fibers [9]. Importantly, it differs from the focal or mass-like uptake more typical of malignancy and from the irregular, localized patterns usually associated with infectious myositis. In this context, PET/CT was pivotal in raising suspicion of muscular sarcoidosis and guiding the diagnostic approach toward histological confirmation [9].

Muscle biopsy remains the gold standard for diagnosis, allowing direct visualization of non-caseating granulomas [2]. However, some authors have noted that granulomatous muscle involvement does not always correlate with clinically active disease [4]. In the present case, the persistence of marked symptoms despite correction of metabolic abnormalities (including hypercalcemia), the recrudescence of symptoms during corticosteroid tapering, and the subsequent sustained improvement after the introduction and up-titration of azathioprine strongly support true active muscular sarcoidosis rather than an isolated histological finding [4].

As mentioned earlier, neurological involvement is reported in approximately 5-15% of patients with sarcoidosis, and peripheral neuropathy accounts for fewer than 20% of these neurological manifestations, with sensory axonal polyneuropathy being among the rarest forms [3,6]. Electromyography was essential to distinguish axonal from demyelinating neuropathy, which would have different therapeutic and prognostic implications.

The absence of pulmonary involvement or lymphadenopathy, features present in up to 90% of systemic sarcoidosis cases [1,10], significantly complicated and delayed diagnosis in this case, underscoring the diagnostic challenge posed by atypical presentations. Although thoracic disease is the most common manifestation, isolated or predominant extrapulmonary sarcoidosis with minimal or absent pulmonary findings is a recognized entity and may lead to diagnostic delay when typical clinical and radiological clues are lacking [11].

## Conclusions

This case illustrates an unusual presentation of sarcoidosis with isolated muscular and peripheral nervous system involvement, in the absence of pulmonary or other common extrapulmonary manifestations. Such atypical manifestations may delay diagnosis and treatment, particularly when initial symptoms are nonspecific and mimic more prevalent conditions. Recognition of characteristic findings, such as diffuse muscular uptake on PET imaging and confirmation through tissue biopsy, is crucial for establishing an accurate diagnosis.

Furthermore, this report emphasizes the importance of close clinical monitoring in patients with sarcoidosis, as disease progression or involvement of new target organs may occur over time. Early identification of neuromuscular involvement allows timely initiation of immunosuppressive therapy, facilitating symptom control, minimizing long-term morbidity, and enabling safe corticosteroid tapering through the use of steroid-sparing agents.
